# Assessing the effectiveness of performic acid disinfection on effluents: focusing on bacterial abundance and diversity

**DOI:** 10.1007/s11356-024-34958-4

**Published:** 2024-09-18

**Authors:** Sadia Bagagnan, My Dung Jusselme, Vanessa Alphonse, Sabrina Guerin-Rechdaoui, Anthony Marconi, Vincent Rocher, Regis Moilleron

**Affiliations:** 1 Laboratoire Eau Environnement Et Systèmes Urbains (Leesu), Univ Paris Est Creteil, Ecole Des Ponts, 61 Avenue du Général de Gaulle, 94000 Créteil, France; 2 Direction de L’Innovation, SIAAP, 92700 Colombes, France

**Keywords:** Performic acid, Wastewater treatment, Disinfection, Microbial diversity, Microbial abundance, Microbial activity

## Abstract

Poorly-treated wastewater harbors harmful microorganisms, posing risks to both the environment and public health. To mitigate this, it is essential to implement robust disinfection techniques in wastewater treatment plants. The use of performic acid (PFA) oxidation has emerged as a promising alternative, due to its powerful disinfection properties and minimal environmental footprint. While PFA has been used to inactivate certain microbial indicators, its potential to tackle the entire microbial community in effluents, particularly resistant bacterial strains, remains largely unexplored. The present study evaluates the efficacy of PFA disinfection on the microbial communities of a WWTP effluent, through microbial resistance mechanisms due to their membrane structure. The effluent microbiome was quantified and identified. The results showed that the number of damaged cells increases with CT, reaching a maximum for CT = 240 mg/L•min and plateauing around 60 mg/L•min, highlighting the optimal conditions for PFA-disinfection against microbial viability. A low PFA level with a 10-min contact time significantly affected the microbial composition. It is worth noting the sensitivity of several bacterial genera such as *Flavobacterium*, *Pedobacter*, *Massilia*, *Exiguobacterium*, and *Sphingorhabdus* to PFA, while others, *Acinetobacter*, *Leucobacter*, *Thiothrix*, *Paracoccus*, and *Cloacibacterium*, showed resistance. The results detail the resistance and sensitivity of bacterial groups to PFA, correlated with their Gram-positive or Gram-negative membrane structure. These results underline PFA effectiveness in reducing microbial levels and remodeling bacterial composition, even with minimal concentrations and short contact times, demonstrating its suitability for widespread application in WWTPs.

**Supplementary Information:**

The online version contains supplementary material available at 10.1007/s11356-024-34958-4.

## Introduction

Insufficiently or partially treated wastewater is likely to harbor pathogenic microorganisms, which can then find their way into the receiving environment via discharges (Drury et al. 2013). As a result, these discharges can impair the quality of receiving waters, which are increasingly frequented by the public for recreational purposes such as bathing (Rhymes et al. 2015). To prevent or mitigate the degradation of the microbiological quality of these waters, the incorporation of a disinfection step in wastewater treatment plant (WWTP) effluents becomes imperative. There are numerous disinfection techniques available, including chlorination, ultraviolet (UV) treatment, and ozonation. However, each technique has its own set of advantages and drawbacks. For example, chlorination, widely acclaimed for its effectiveness against pathogenic bacteria, has the major drawback of producing harmful disinfection by-products (DBPs) known to be toxic and potentially carcinogenic, teratogenic, and mutagenic (Lin et al. 2021; Xu et al. 2022). The possibility of implementing advanced oxidation processes based on non-specific oxidation mechanisms, such as ozonation and UV radiation, represents an option that has met with variable success. However, their large-scale application implies high operating costs due to their high energy demand (Sun et al. 2023). These constraints make these disinfection techniques less attractive for implementation in WWTPs.

In order to identify an effective disinfectant with minimal or no disinfection by-products formation, nor non-toxic degradation products for the aquatic environments, and cost-effectiveness, researchers are exploring the use of peracids, including peracetic acid (PAA) and performic acid (PFA). Initially, peracetic acid attracted attention for wastewater disinfection due to its strong oxidation capabilities, particularly in countries such as the UK (Falsanisi et al. 2006). Its effectiveness covers a broad spectrum of pathogenic microorganisms, ranging in sensitivity from bacteria to protozoa, classified as follows: bacteria > viruses > spores > cysts (Acosta et al. 2022; Gesto et al. 2018). However, a major drawback of this approach lies in potential microbial reviviscence and proliferation. The acetic acid produced by PAA degradation serves as a source of carbon for microorganisms, promoting their growth and proliferation (da Silva et al. 2020). PFA has now emerged as an alternative to peracetic acid. Possessing robust oxidizing properties close to those of peracetic acid, PFA is synthesized from formic acid (HCOOH) catalyzed by sulfuric acid and hydrogen peroxide H_2_O_2_ (Gehr et al. 2009). Its synthesis reaction generates heat (Ragazzo et al. 2013), and the PFA produced must be used quickly after synthesis due to its instability (Gaikwad et al. 2017). Historically used as a bleaching agent in paper pulp production, replacing chlorinated chemicals (Brasileiro et al. 2001; Lang et al. 2018), PFA is also finding application as a disinfectant in the medical field, due to its effectiveness against pathogenic microorganisms. Recent studies have extensively evaluated its efficacy in treating effluents at different stages of treatment: primary (Gehr et al. 2009), secondary (Ragazzo et al. 2013), or tertiary (Luukkonen et al. 2015) and even for combined sewer overflow discharges (Chhetri et al. 2014). These disinfection trials have targeted specific microorganisms, including fecal indicator bacteria such as *Escherichia coli*, intestinal enterococci, and other pathogens, such as the bacterial genera *Clostridium*, *Aeromonas*, *Salmonella*, spore-formers such as *Cryptosporidium* and cysts such as *Giardia* (Gehr et al. 2009; Karpova et al. 2013).

In numerous experiments, PFA has demonstrated remarkable efficacy against these pathogenic microorganisms at low concentrations and short contact times (Chhetri et al. 2014; Gehr et al. 2009; Luukkonen et al. 2015; Ragazzo et al. 2013). The main mode of PFA action is the disruption of bacterial cells by damaging their cell walls, resulting in a loss of protective capacity and the release of internal cell contents (Ding et al. 2023; Lin et al. 2023). In addition, the PFA efficiency can vary depending on the treatment level of these discharges. For example, the removal of 1 log of *Enterococci* requires a concentration of 1 mg/L for primary effluents vs. 0.5 mg/L for secondary effluents, with a maximum contact time of 20 min (Gehr et al. 2009). In addition, this efficiency can vary depending on the type of microorganisms. For the same concentration and contact time, the reduction of *E. coli* is greater than that of *Enterococci*. In particular, for pathogens such as *E. coli*, an abatement of between 2.2 and 4 logs was achieved with PFA doses ranging from 0.9 to 1.2 mg/L in a contact time of less than 10 min (Ragazzo et al. 2013). In the same study (Ragazzo et al. 2013), at similar concentrations and contact times, *Enterococci* elimination ranged from 0.7 to 3.2 logs. Furthermore, as part of its proven biocidal action, PFA breaks down into formic acid, hydrogen peroxide, and CO_2_ (Santacesaria et al. 2017), all of which are not toxic to the aquatic environment (Gehr et al. 2009). Notably, no significant general toxicity effects were observed in bacteria, fungi (*Septoria tritici*) and yeasts (*Saccharomyces cerevisia*), which serve as biological models for the receiving waters of effluents disinfected with PFA (Rocher and Azimi 2021). Furthermore, a comparison between peracetic acid and performic acid shows a greater bacterial inactivation capacity for PFA (Campo et al. 2020; Chhetri et al. 2014). As a result, PFA represents an attractive option for water treatment plant managers. Previous experiments involving performic acid have mainly targeted specific microorganisms. However, wastewater contains many other microorganisms, including genera such as *Campylobacter*, *Pseudomonas*, *Shigella*, and *Aeromonas* (Chahal et al. 2016), all of which are potential pathogens. Hence, the present study, carried out as part of the MeSeine Innovation research program, therefore aimed to assess the effectiveness of PFA by examining the entire effluent microbiome. More specifically, our aim was to study the resistance of indigenous microorganisms in effluents to PFA action. The microbial community will be analyzed according to two well-defined aspects: abundance and diversity.

## Material and methods

### Effluent collection

Effluent samples were taken at the outlet of the Seine Centre (SEC) WWTP in Colombes (France), near Paris, in October 2021. At this WWTP, the wastewater comes from a population of around one million inhabitants. The WWTP treats a daily volume of water of around 240,000 m^3^ in dry weather and up to 404,800 m^3^ in wet weather conditions. A sequence of treatment techniques is applied before the treated effluent is discharged into the Seine River: physicochemical laminar settling followed by biological treatment using a bio-filtration unit to eliminate carbon and nitrogen pollution. Once collected, the effluent was stored at a temperature of − 20 °C until the disinfection tests were carried out.

### PFA synthesis

PFA was synthesized according to the protocol detailed in (Rocher and Azimi 2021). The first step involved preparing catalyzed formic acid by adding 0.96 g sulfuric acid (98%, Aldrich) to 7.49 g formic acid (99%, Aldrich) over ice. The second step involved adding of 1,370 mL H_2_O_2_ (50% w.t., Aldrich) to 1260 mL formic acid solution to generate performic acid (PFA) in an ice bath. This mixture is slowly stirred for 90 min under cold conditions. Once the 90 min have elapsed, a PFA solution is ready for use. The PFA concentration was measured by iodometric titration. To this end, 83.4 µL of this mixture was mixed with 25 mL of H_2_SO_4_ (1 M) solution and titrated to determine the concentrations of H_2_O_2_ and PFA. H_2_O_2_ titration was carried out with potassium permanganate solution (KMnO_4_, 0.02 M, Aldrich). Excess potassium iodide was then added and oxidized by PFA. Iodine was then titrated with sodium thiosulfate (Na_2_SO_3_, 0.02 M, Aldrich). The PFA mass concentrations obtained range from 8 to 11%. The PFA solution was stored at 4 °C and titrated in triplicate just before use to confirm its concentration.

### Experimental design

Disinfection was carried out in a laboratory-scale microcosm. This was set up using a laboratory fermenter (Minfor2, France) to maintain a constant temperature (18 °C). In the microcosm, 4 L of effluent were continuously stirred at 200 rpm. During agitation, performic acid was gradually added to target concentrations of 0.8, 2, and 4 mg/L. For each concentration, samples were collected at different contact times (0, 5, 10, 15, 30, 45, and 60 min). The experiment was carried out for those three PFA concentrations in triplicate. For a given contact time, immediately after sampling, Na_2_SO_3_ was added to reach a final concentration of 0.02 M. The Na_2_SO_3_ concentration then exceeded the concentrations of performic acid and H_2_O_2_ by a factor of 62, ensuring complete quenching of their oxidation potential (Feng et al. 1995). PFA-treated samples were stored at − 20 °C prior to analysis. CT (mg/L•min) values were calculated by multiplying concentration (in mg/L) by contact time (in minutes).

### Microbial community analysis

#### Bacterial abundance measurement

Bacterial cells in all samples, including those untreated with PFA and those treated at three concentrations (0.8, 2, and 4 mg/L) for seven contact times (0, 5, 10, 15, 30, 45, and 60 min), were measured by flow cytometry using the BactoSense-automated flow cytometer (FCM) instrument (bNovate, Switzerland). This technique is commonly used to measure bacterial abundance in liquid environmental samples. The BactoSense works on the principle of employing double labelling with two fluorochromes: SYBR Green I and propidium iodide (PI). SYBR Green I, a dye, penetrates all bacterial cells irrespective of membrane integrity, enabling all cells to be quantified. On the other hand, PI penetrates only cells with damaged membranes, enabling quantification of damaged cells (Grégori et al 2001). This double-staining technique simultaneously quantifies total, intact, and damaged cells. For the measurement, an automated extraction of 260 µL of sample was carried out, 90 µL of which was labelled with the fluorochromes. The stained samples were then incubated at 37 °C for 10 min prior to cell counting. Samples were measured in triplicate. To avoid contamination, thorough cleaning was carried out between sample measurements. The raw FCM data files were then analyzed using customized software that enabled batch processing of the large datasets generated in this study. In brief, FCM gates are built to separate bacterial cell signals (total cell concentration, TCC) from background signals and to differentiate between intact cells from cells with altered membranes. Accordingly, the results provided the numbers of total cells, intact cells, and damaged cells as Excel files.

#### Bacterial diversity assessment

##### High-throughput sequencing

Untreated and PFA-treated samples were analyzed at three concentrations (0.8, 2, and 4 mg/L) and for three contact times (0, 10, and 60 min) to assess their bacterial diversity. To collect the microorganisms, the untreated and PFA-treated samples (500 ml) were filtered through a sterilized vacuum pump system with 0.22 µm Whatman nitrate-cellulose filters (Sartorius, France) under a laminar flow hood (BIOII, ADS Laminaire, France). After filtration, the filters containing the microorganisms were placed in labelled sterile Petri dishes under a laminar flow hood for 30 min to dry. DNA was extracted from these filters using the DNeasy PowerWater Kit (QIAGEN) according to the manufacturer’s instructions. Library preparation and high-throughput sequencing were performed on an Illumina MiSeq platform by Eurofins Genomics (Germany) according to their standard protocols. The V3–V4 variable region was amplified with the forward primer (5′-TAC GGG AGG CAG CAG-3′) and the reverse primer (5′-CCA GGG TAT CTA ATC C-3′). Illumina raw sequences have been deposited in the NCBI database under the BioProject ID: PRJNA666519.

##### Data processing

The sequence datasets output by Eurofins were bioinformatically processed using the FROGS analysis pipeline available on INRAE’s Galaxy platform, the steps of which are described by Escudié et al. (2018). As a first step, the sequences were cleaned up by eliminating those that were too short or had too many ambiguous bases (errors). These cleaned sequences were then grouped into clusters from which chimeras were removed in the third step. A filtration step ensured that only clusters containing more than 0.0005% of the sequences were retained. This treatment produced taxonomically affiliated operational taxonomic units (OTUs) that were taxonomically affiliated using the Blast 16-SILVA-138 database. Diversity indices such as Shannon index and Simpson richness were calculated using the R package Phyloseq in FROGS (FROGSSTAT). The Simpson index represents the uniformity, while microbial diversity was assessed by the Shannon index. Based on data obtained from 16S RNA sequencing, it was possible to predict the potential functions of the various bacterial communities identified using PICRUSt (Phylogenetic Investigation of Communities by Reconstruction of Unobserved States). The OTU table was used as an input file for PICRUSt, and a correlation was established between phylogeny and the functions. The abundance of the various predicted genes identified was analyzed using KEGG (Kyoto Encyclopedia of Genes and Genomes).

### Statistical analyses

Variations between untreated and PFA-treated effluent samples for the parameters of number of damaged cells and the relative abundance of microbial groups were assessed by one-way ANOVA (Fisher’s post hoc test) with a 95% confidence interval, using the R-3.6.1 software (France). Correlation tests between genus-level bacterial diversity data and predicted function data were performed using the XLSTAT software (Addinsoft, USA).

## Result and discussions

### Evaluating the number of damaged bacterial cells in the effluent after PFA disinfection

**Fig. 1:**
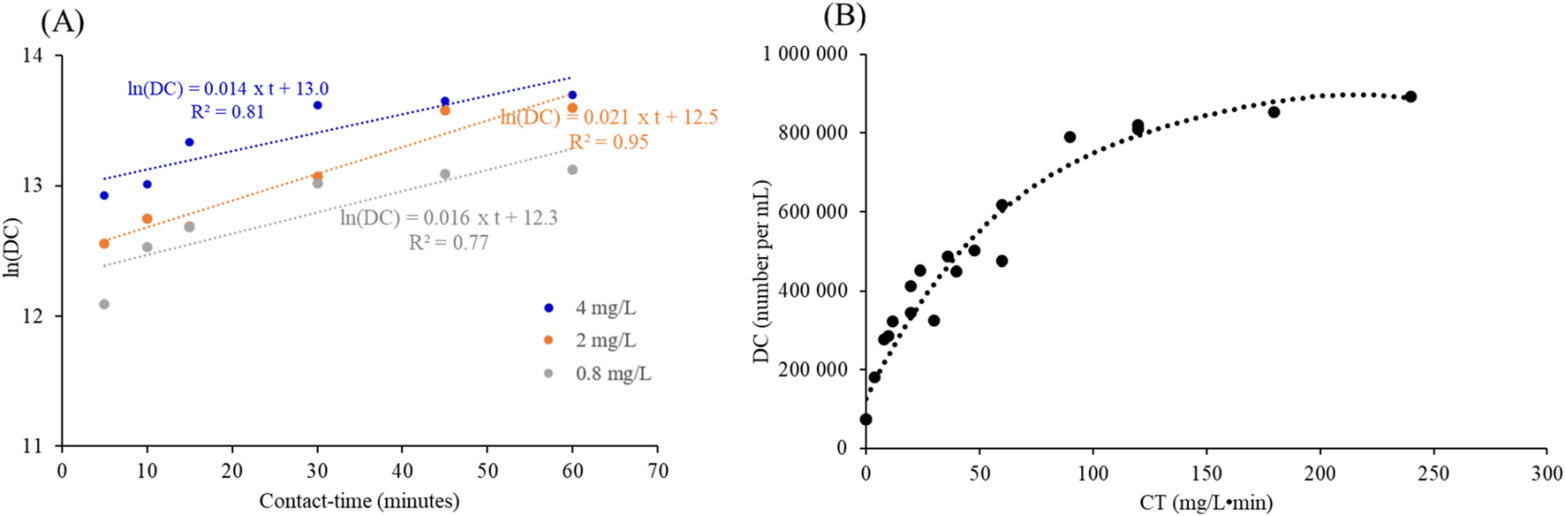
ln(DC) (number of damaged cells per mL) with contact time (min) at different concentrations of PFA (**A**) and DC relative to CT (mg/L•min) (**B**)

The number of damaged cells in the WWPT effluents for three PFA concentrations of 0.8, 2, and 4 mg/L and for different contact times (0, 5, 10, 15, 30, 45, and 60 min) was monitored using a flow cytometry technique with double labelling of viable and damaged cells. This approach proves more appropriate for monitoring the PFA effect on bacterial abundance, as the PFA action disrupts bacterial cells by damaging their cell walls, resulting in loss of protective capacity and release of internal cell contents. Figure 1A therefore shows the number of damaged cells (DC) as a function of time for the three PFA concentrations, while Fig. 1B shows the evolution of the number of damaged cells with CT (expressed in mg/L•min), i.e., taking into account the combination of PFA concentration and contact time. Figure 1A shows that the DC number increases with time for all PFA concentrations. The highest DC number was observed for a PFA concentration of 4 mg/L after 60 min of contact time (i.e., a CT of 240 mg/L•min). These results highlighted the optimal conditions of PFA in disinfecting WWTP effluents. Figure 1B highlights the importance of the contact time, since the DC number increases with CT. However, this number of damaged cells stabilized, reaching a plateau around 60 mg/L•min, remaining at around 60% until 240 mg/L•min. The value of 60 mg/L•min represents the peak of activity, in agreement with the results of Wang et al. (2023b), where a value of 60 mg/L•min demonstrated more effective inactivation *E. coli*, reaching an efficiency rate of 99%. Therefore, for the WWTP operators, this means that the same level of disinfection can be achieved by increasing either the PFA concentration or the contact time.

### Characterization of the composition of bacterial community in untreated effluent

**Fig. 2:**
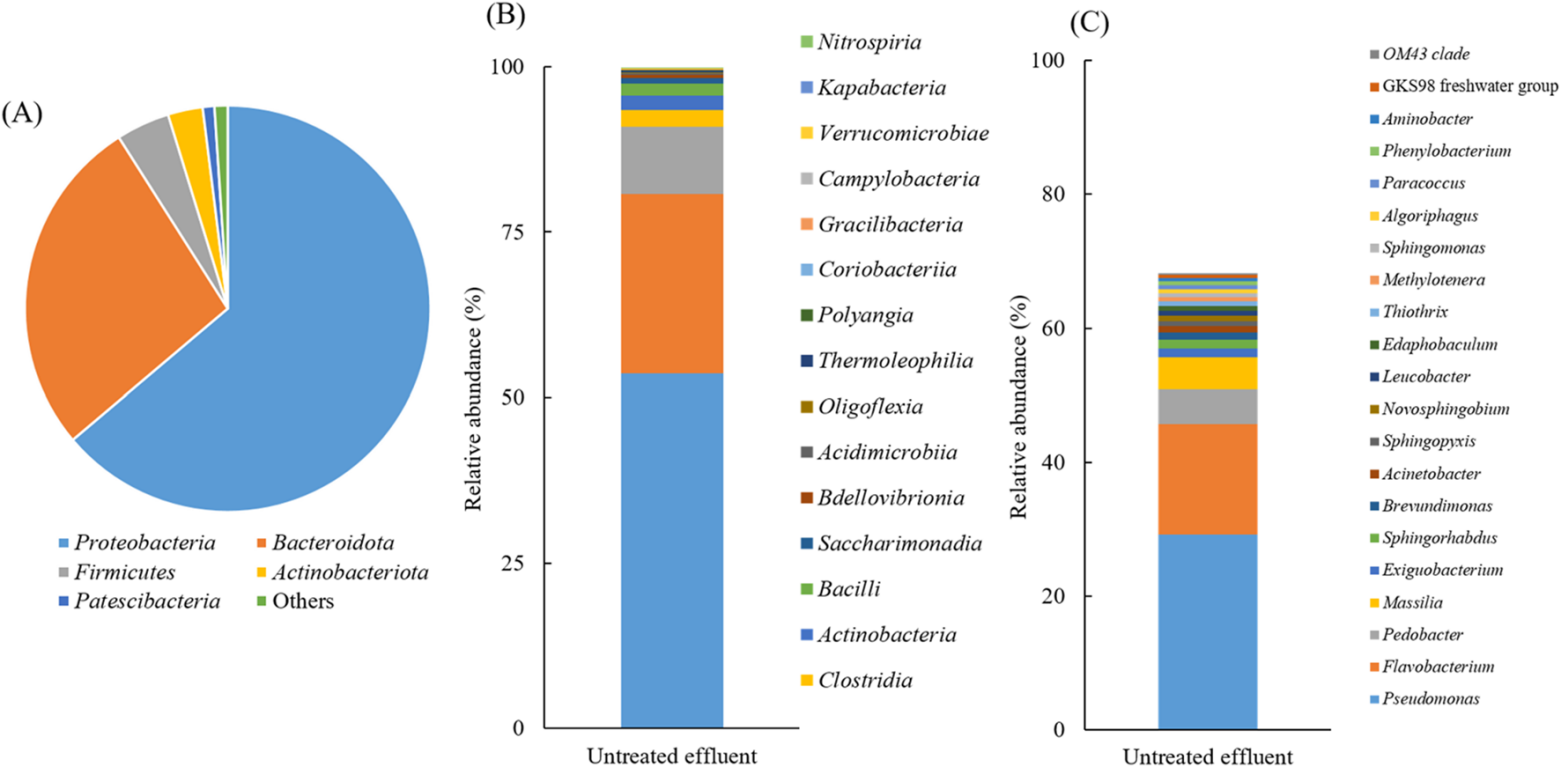
Relative abundance of bacterial community in the untreated effluent at the level of **A** phylum, **B** class, and **C** genus

Firstly, the bacterial communities present in the untreated effluent were characterized. These results served as a basis for future comparison, before assessing changes in the bacterial community resulting from PFA disinfection. The dominant phyla were *Proteobacteria* (63.8%), *Bacteroidota* (27.1%), *Firmicutes* (4.3%), *Actinobacteriota* (2.7%), and *Patescibacteria* (0.9%) (Fig. 2A). These identified phyla are commonly found in the microbiome of WWTP effluents, as previous studies have indicated (Zhang et al. 2019; Wang et al. 2023a). To assess the effectiveness of PFA on pathogens and its action on membrane cells, this section presents and discusses the bacterial groups classified according to these two characterizations. At the class level, seven predominant classes were observed, including *Gammaproteobacteria* (53.7%) and *Alphaproteobacteria* (10.1%) belonging to the phylum *Proteobacteria*, *Bacteroidia* of the phylum *Bacteroidota* (27.1%), *Clostridia* (2.5%) and *Bacilli* (1.8%) belonging to the phylum *Firmicutes*, *Actinobacter* (2.3%) from the phylum *Actinobacteriota*, and *Saccharimonadia* (0.9%) from the *Patescibacteria* phylum (Fig. 2B). Further analysis of the bacterial genus showed that the dominant genes were *Pseudomonas* (29.2%), *Massilia* (4.8%), *Sphingorhabdus* (1.3%), and *Actinetobacter* (0.9%), belonging to the *Gammaproteobacteria* class; *Brevundimonas* (1.1%) and *Sphingomonas* (0.6%), belonging to the *Alpha*p*roteobacteria* class; *Flavobacterium* (16.5%) and *Pedobacter* (5.2%) belonging to the *Bacteroidia* class (phylum *Bacteroidota*), *Exiguobacterium* (1.3%) from the *Clostridia* class (phylum *Firmicutes*) (Fig. 2C). The bacterial cell envelope is a complex, multilayered structure that protects bacteria from hostile environments. Most bacterial envelopes fall into two groups: Gram-negative and Gram-positive. Gram-negative bacteria have a thin peptidoglycan cell wall, while Gram-positive bacteria have much thicker layers of peptidoglycan, which can provide protection against the action of PFA. In the untreated effluent, *Pseudomonas* (29.2%), a Gram-negative bacterium known for its resistance to antimicrobial treatments due to the presence of three exopolysaccharides (Psl, Pel, and alginate) in its membrane structure (Chino et al. 2017), was present. Other Gram-negative bacteria identified were *Flavobacterium* (16.5%) (Wang et al. 2016), and *Sphingorhabdus* (3.2%) (Jogler et al. 2013). In addition, several potential pathogens causing infections in immunocompromised individuals were found in effluent such as *Pseudomonas aeruginosa*, classified in the genus *Pseudomonas* and *Massilia timonae*, belonging to the genus *Massilia*. In addition, pathogens such as *Acinetobacter*, *Brevundimonas*, and *Flavobacterium* were present in untreated effluent, causing nosocomial infections in vulnerable hospital patients (Ryan and Pembroke 2018). Similarly, *Klebsiella* sp. and *Escherichia*-*shigella*, responsible for gastroenteritis (Okoh et al. 2007), were identified in untreated effluent at low abundances of 0.001% and 0.002%, respectively. Consequently, these pathogens were included in the follow-up disinfection tests.

### Changes in bacterial composition in the treated effluents with CT (mg/L•min) compared to untreated effluent

**Fig. 3:**
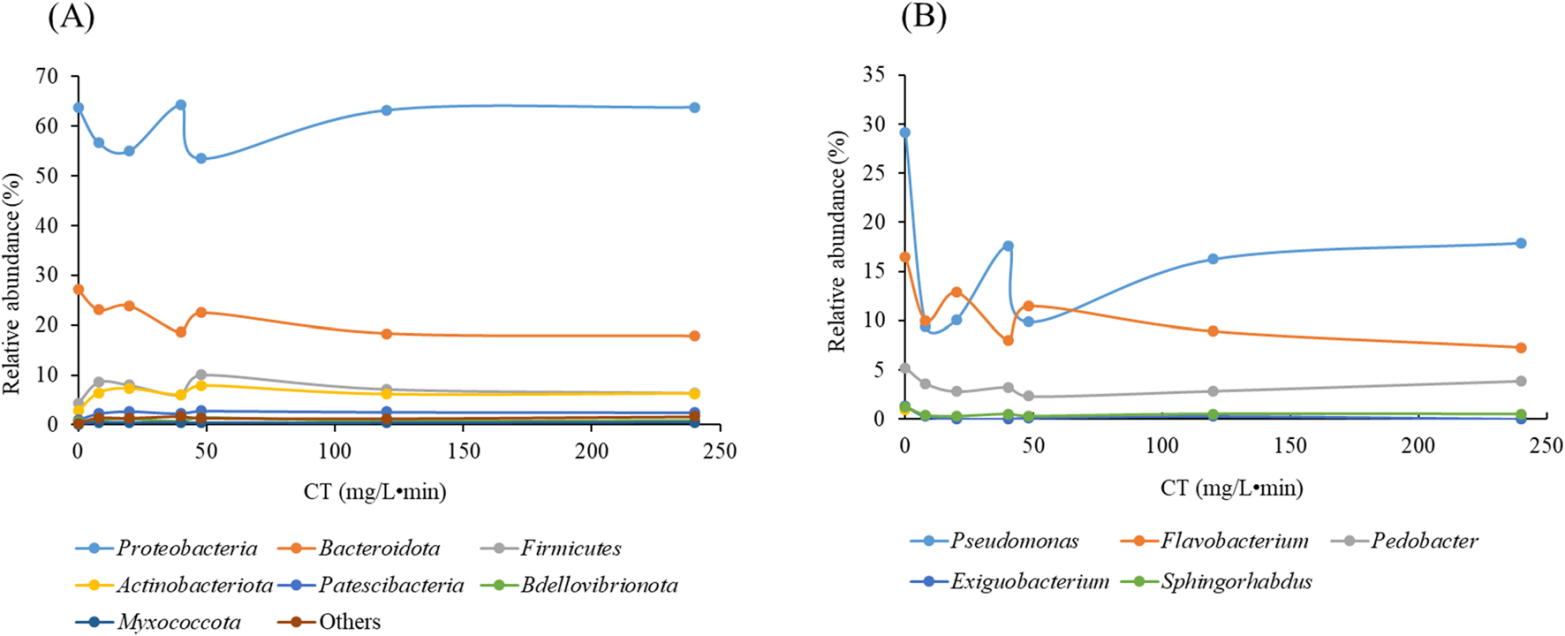
Changes in the relative abundance of the bacterial community during PFA disinfection: at the level of phylum (**A**) and PFA-sensitive bacterial strains (**B**)

Relative abundances of bacterial composition at phylum and genus level were monitored at different CT values during the disinfection treatment. Figure 3A summarizes the changes that occurred during PFA disinfection in terms of the relative abundance of bacterial community at the level of the main phyla. For CT below 48 mg/L•min, fluctuations were observed in the relative abundance of all phyla. These fluctuations differed from phyla to phyla, making it difficult for treatment plant operators to understand how PFA affects these phyla at low CT (i.e., below 48 mg/L•min). However, for a CT higher than 60 mg/L•min, a plateau was reached for all the phyla, confirming previous observations concerning the number of damaged cells. This is in agreement with previous results (Karpova et al. 2013) since a CT of 60 mg/L•min is considered the optimal dose for disinfection. In detail, for *Proteobacteria*, PFA action led to a decrease in their relative abundance from 63.8% before treatment (CT = 0) to around 55%, with the exception of the CT40, for which the relative abundance reached 64.2%. For high CTs (> 60 mg/L•min), the relative abundance of *Proteobacteria* was close to that of the untreated effluent. This is explained by the fact that the relative abundance of *Proteobacteria* was associated with an increase in the relative abundance of *Alphaproteobacteria*, one of the main classes of this phylum. The relative abundance of *Alphaproteobacteria* increased from 10.2% before treatment to around 15% for high CTs. In general, members of the *Alphaproteobacteria* class are gram-negative bacteria (Gupta 2000), and are more sensitive to the action of PFA. The increase in this bacterial group could be explained by another pathway for their ability to resist to PFA, apart from their negative membrane, or because the relative abundance of other groups is decreasing, leading to an increase in this group. Conversely, for *Bacteroidota*, a different trend was observed. The relative abundance of *Bacteroidota* decreased with increasing CT. Initially at 27.1%, the relative abundance of *Bacteroidota* decreased to around 20% for higher CTs. This trend suggests that members of this phylum are sensitive to the action of PFA, potentially due to their Gram-negative membrane structure (Hudson and Egan 2022). This observation is consistent with previous results (Ding et al. 2023), demonstrating that the PFA efficacy is higher against Gram-negative bacterial cells. Furthermore, the higher the concentration, the stronger the action of the PFA on *Bacteroidota*. This rapid action is in line with previous results (Luukkonen et al. 2015), where *E.coli* showed a rapid 2.9 log reduction after 1 min and reached a 3.5-log reduction after 60 min of contact time. Finally, the phyla *Firmicutes*, *Actinobacteriota*, and *Patescibacteria* showed an increase in their relative abundance for high CTs. *Firmicutes* increased from 4.3% before treatment to 6.0% for high CTs. This increase in the relative abundance of *Firmicutes* is linked to the increase in *Clostridia* class. The behavior of *Firmicutes* could be elucidated by considering the characteristic membrane structure of bacteria in this phylum, in relation to the mechanism of action of PFA. Many bacteria in this phylum feature the Gram-positive membrane, characterized by a membrane with a thick layer of peptidoglycan, which provides a certain level of protection (Pang et al. 2016). This characteristic is particularly marked in the *Clostridia* class, known for its resistant to PFA action. In addition to their membrane structure, these bacteria have the ability to produce endospores, enabling them to survive even under harsh conditions (Kaldhusdal and Jordan 2008). Notably, this class of bacteria has shown resistance to disinfectants such as chlorine (Pang et al. 2016) and peracetic acid (Eramo et al. 2017). In contrast, the relative abundance of *Bacilli* remained constant during all experimental phases, irrespective of disinfection. Like *Clostridia*, bacteria belonging to the *Bacilli* class possess an exclusive Gram-positive with a robust membrane structure (Ludwig et al. 2015). This characteristic could explain their resistance to PFA disinfectant action. As for *Actinobacteriota*, their relative abundance increased from 2.7% before treatment to 6.0% for high CTs. This observation suggests resistance to PFA. *Actinobacteria* classes are capable of forming exo-spores to protect themselves in the presence of unfavorable external conditions such as disinfectants (Beskrovnaya et al. 2021). Like *Clostridia*, *Actinobacteria* are resistant to chlorine (Li et al. 2020; Wang et al. 2023a).

**Fig. 4:**
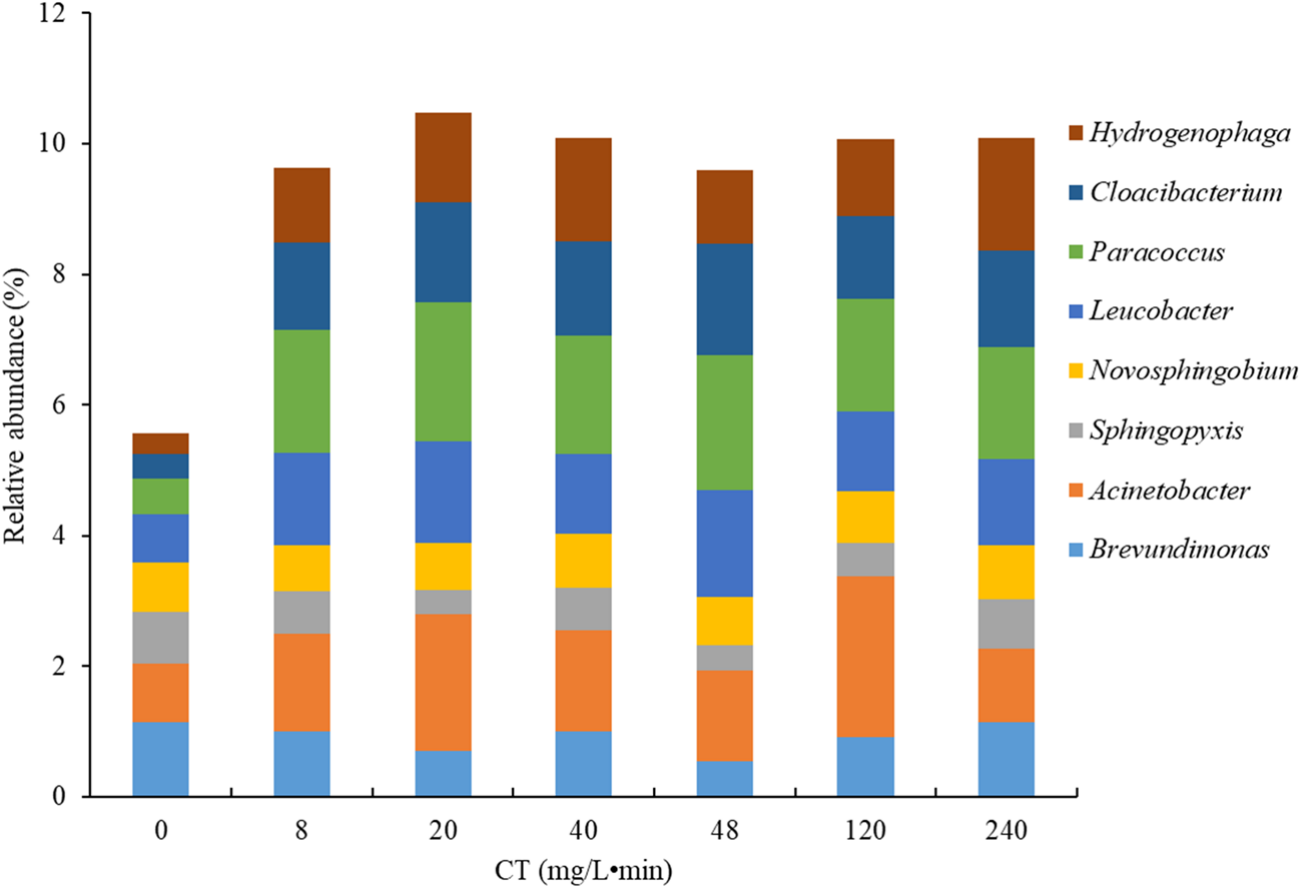
Relative abundance of PFA-resistant bacterial community at the genus level during the disinfection process

To better understand the impact of PFA, the disinfection effect of PFA was evaluated at the bacterial genus level (Fig. 3B). *Pseudomonas*, the most abundant bacterial genus identified in the untreated effluent, showed a decrease in relative abundance during the treatment process. Their relative abundance dropped from 29.2% before treatment up to around 17.0% at high CTs. This reduction reflects the impact of PFA on certain bacteria of this genus; although they are generally known for their resistance to various disinfection methods such as chlorine, UV, and ozone (Wang et al. 2020). The increase in *Pseudomonas* abundance could be explained in part by the elimination of certain other bacteria. This indicates that, in addition to *Pseudomanas*, other bacteria are unable to resist the effect of PFA. As shown in Fig. 3B, there is a correlation between the increase in *Pseudomonas* abundance and a simultaneous decrease in *Flavobacterium* abundance. In addition, the impact of PFA was observed on the genera *Flavobacterium*, *Pedobacter*, *Massilia*, *Exiguobacterium*, and *Sphingorhabdus*, resulting in a decrease in their abundance in the treated effluent. It should be noted that all these bacterial genera are Gram-negative (Jogler et al. 2013; Li et al. 2017), which could explain their sensitivity to the action of PFA. Conversely, certain bacterial genera show increased relative abundance during PFA disinfection, notably *Acinetobacter*, *Leucobacter*, *Thiothrix*, *Paracoccus*, *Cloacibacterium*, and *Hydrogenophaga* (Fig. 4). These genera, which dominated in the untreated effluent, were among the dominant taxa in the treated effluent after PFA addition. This trend suggests their resistance to PFA. Notably, some bacteria in these resistant genera are Gram-negative (such as *Acinetobacter*, *Paracoccus Cloacibacterium*, and *Hydrogenophaga*) (Bates and King 2021; Chung et al. 2007), while one genus shows both Gram-negative and positive variability (*Thiorix*) (Unz and Head 2015). For some genera, the abundance has remained stable, with no downward or upward trend; this is the case for *Brevundimonas*, *Novosphingobium*, *Methylotenera*, and *Sphingomonas*. Bacteria in these genera are Gram-negative. These observations concerning the membrane structure of PFA-sensitive and PFA-resistant bacteria indicate a more pronounced effect of PFA on Gram-negative bacteria than on Gram-positive bacteria. Gram-positive bacteria generally have thicker cell walls, ranging from 20 to 80 nm thick (compared with 1.5 to 10 nm for Gram-negative bacteria), giving them a certain resistance to disinfection (Mai-Prochnow et al. 2016). However, some Gram-negative bacteria show PFA-resistance despite these factors. When assessing the effectiveness of PFA against pathogenic microorganisms, it is evident that among the potential pathogenic microorganisms identified, *Pseudomonas*, initially dominant in the untreated effluent, showed a reduced but persistent presence in the treated effluent. A decrease in the relative abundance of *Massilia* was observed at certain CT values, as some *Massilia* species, such as *Massilia timonae*, are known to cause infections in immunocompromised individuals (Lindquist et al. 2003). Another observation concerning *Flavobacterium* was that, despite its resistance to chlorine treatment, this genus showed a decreasing abundance during the disinfection process. Other pathogens such as *Acinetobacter* and *Brevundimonas* were identified in the treated effluent, illustrating their ability to resist PFA. Among the bacteria monitored to assess ecological status, *Mycobacterium*, *Enterococcus*, and *Streptococus* were detected in minimum proportions of 0.02, 0.01, and 0.11%, respectively. No reduction in relative abundance was observed for any of these bacteria during PFA disinfection. Other pathogenic bacteria such as *Klebsiella* and *Escherichia-Shigella*, known to cause gastroenteritis (Okoh et al. 2007), were found in the untreated effluent with abundances of 0.001 and 0.002%, respectively. However, complete removal for them was achieved at a CT of 8 mg/L•min for *Klebsiella* and 20 mg/L•min for *Escherichia-Shigella*.

### Changes in functional profiles

**Fig. 5:**
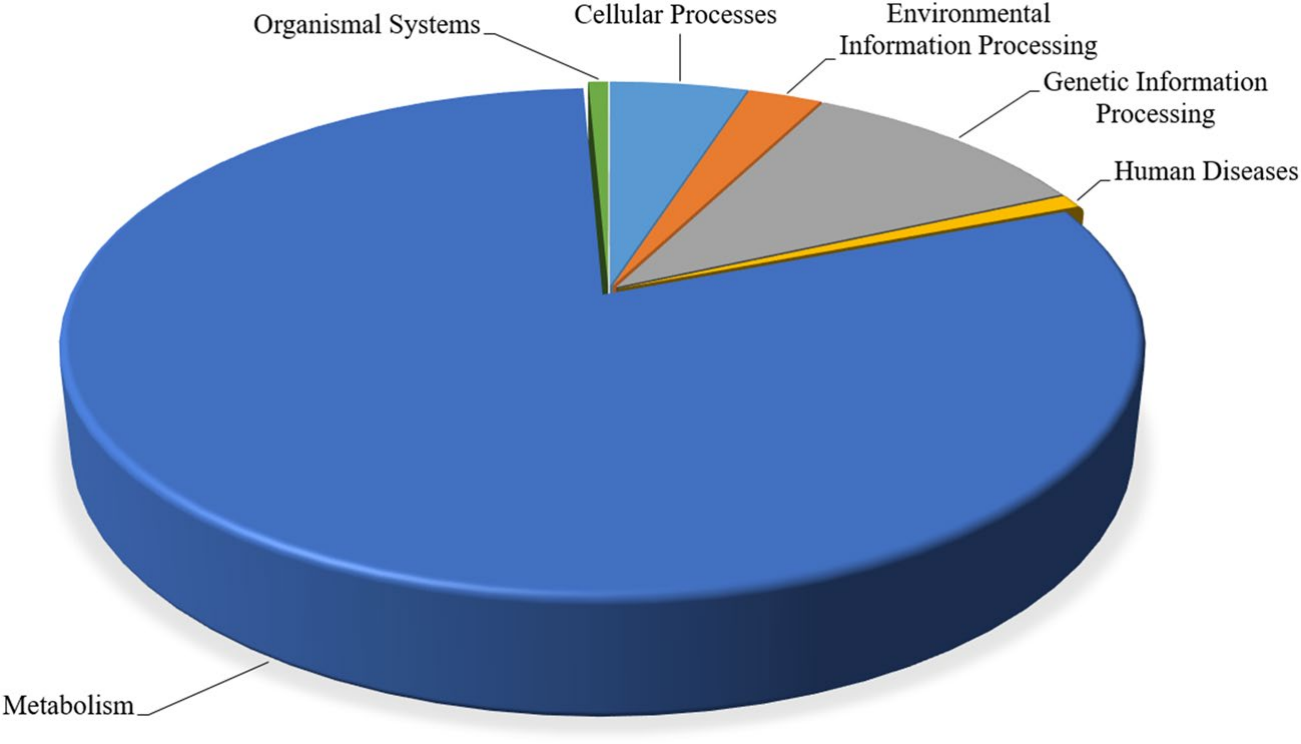
Relative abundance of KEGG pathway groups at the level 1 in untreated effluent

Functional gene predictions for bacterial communities were carried out using the PICRUSt software, with the aim of discerning different functional profiles within the sample. In the untreated effluent, functional profiles related to KEGG pathways were classified into six main functional groups at level 1: metabolism (80.2%), genetic information processing (10.6%), cellular processes (4.96%), environmental information processing (2.63%), human diseases (0.86%), and organizational systems (0.71%) (Fig. 5). At level 2, function classification revealed 11 pathways for metabolism, 4 pathways for genetic information processing, 2 pathways for environmental information processing, 5 pathways for cellular processes, 4 pathways for organ systems, and 6 pathways for human diseases. Among the 32 KEGG level 2 pathways, the relative abundance of replication and repair, amino acid metabolism, carbohydrate metabolism, energy metabolism, lipide metabolism, and xenobiotic degradation was the highest in all samples including those not treated with PFA at different CT values (Table S1, supplementary data). The impact of PFA on gene prediction was assessed for the effluent treated at six CT values, corresponding to three concentrations (0.8, 2, and 4 mg/L) and at two contact times (10 and 60 min). These PFA-treated samples were compared with their untreated counterparts. At KEGG level 1, no clear upward or downward trend in gene abundance was observed for any of the six groups, regardless of whether the effluents were treated or not. Further analysis at level 2 revealed specific changes: a decrease in the relative abundance of genes in the pathway of glycan biosynthesis and cofactor and vitamin metabolism, and an increase in genes related to the pathway of biodegradation and xenobiotic metabolism (Table S1).

**Fig. 6:**
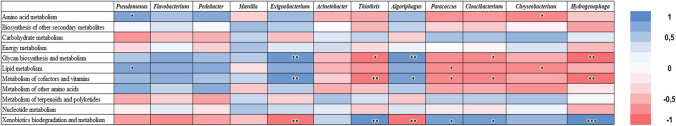
Pearson correlation between bacterial diversity at genus level and predicted function at level 1

In Fig. 6, positive Pearson correlations were identified between the glycan biosynthesis and metabolism pathway, the cofactor and vitamin metabolism pathway, and the relative abundance of the genera *Exiguobacterium* and *Algoriphagus*. Similarly, another correlation was found between xenobiotic biodegradation and the metabolism pathway, and the relative abundance of two bacterial strains: *Thiothrix* and *Hydrogenophaga*. The decrease in the xenobiotic biodegradation and the metabolism pathway corresponded to the reduction in the relative abundance of these bacterial strains, as indicated above, suggesting the involvement of these bacterial genera in these biological processes. This result is consistent with previous research indicating a potential role of certain strains of *Exiguobacterium* in glycan biosynthesis and metabolism (Delegan et al. 2021). The observed decrease of *Exiguobacterium* in PFA-treated effluents provides a plausible explanation for this effect.

In addition, attention was paid to the abundance of genes linked to human diseases and their variations during disinfection tests (Table S2, supplementary data*)*. Six classes (level 2) were considered: cancer, cardiovascular diseases, metabolic diseases, immune system diseases, infectious diseases, and neurodegenerative diseases, with neurodegenerative diseases being the most abundant. Among the level-3 functions observed in treated effluents, epithelial cell signaling in *Helicobacter pylori* infection (infectious disease: bacterial), African trypanosomiasis (infectious disease: parasitic) and amyotrophic lateral sclerosis (neurodegenerative disease) were the most prevalent. Interestingly, a decrease in the relative abundance of these functional groups was observed during PFA disinfection. The presence of bacterial communities associated with cancer, viral myocarditis (cardiovascular), toxoplasmosis, Alzheimer’s disease, and Parkinson’s disease pathways is also noteworthy. Moreover, these functional genes are commonly detected in WWTP effluents (Zhao et al. 2020) and even in drinking water (Jiang et al. 2022). It is essential to further investigate the predicted function of genes associated with human disease. This investigation is crucial, as a decline in these diseases due to the action of PFA would enable us to draw conclusions about the effectiveness of PFA treatment on effluents. However, no significant change in relative abundance was observed in these functional groups associated with human diseases, based on the conditions investigated regarding PFA concentration and contact time in the present study. This observation may be explained by the possibility that PFA acts primarily on membrane cells, as indicated by the greater abundance of damaged cells in the PFA-treated samples than in non PFA-treated samples, mentioned above.

## Conclusion

The aim of this study was to evaluate, for the first time, the effectiveness of PFA disinfection on the microbial communities of WWTP effluents, and not only on *E. coli* and total coliforms, as it is generally the case. First, effluent bacterial communities were characterized, with the dominant taxa identified at both phyla level (*Proteobacteria*, *Bacteroidota*, *Firmicutes*, *Actinobacteriota*, and *Patescibacteria*) and genus levels (*Pseudomonas*, *Massilia*, *Sphingorhabdus*, *Actinetobacter*, *Brevundimonas, Flavobacterium, Pedobacter*, and *Exiguobacterium*). Secondly, the effectiveness of PFA disinfection on microbial viability was demonstrated even at the lowest CT value of 4 mg/L•min, with the highest efficacy observed at a CT of 240 mg/L•min. Among the different contact times and concentrations tested, some bacterial genera showed sensitivity to PFA, including *Flavobacterium*, *Pedobacter*, *Massilia*, *Exiguobacterium*, and *Sphingorhabdus*, while others showed resistance, such as *Acinetobacter*, *Leucobacter*, *Thiothrix*, *Paracoccus*, *Cloacibacterium*, and *Hydrogenophaga*. Prediction of metagenomic functions using PICRUSt revealed that metabolism remained the dominant function after PFA disinfection for all effluents. Given these results, the use of PFA in WWTP appears to be a promising option for disinfection.

## Supplementary Information

Below is the link to the electronic supplementary material.

Supplementary file1 (DOCX 38 KB)

## Data Availability

The sequences have been deposited in the NCBI database under the BioProject ID: PRJNA666519.
