# Supplementary material for: Assessing the effectiveness of performic acid disinfection on effluents: focusing on bacterial abundance and diversity
Source: Environ Sci Pollut Res Int. 2024 Sep 18;31(48):58252–62. doi: 10.1007/s11356-024-34958-4 (PMC11467000; doi:10.1007/s11356-024-34958-4)
Supplement: Supplementary file 1 — Supplementary file1 (DOCX 38 KB) [file 11356_2024_34958_MOESM1_ESM.docx]

**Supplementary material**

**Assessing the effectiveness of performic acid disinfection on effluents: focusing on bacterial abundance and diversity**

Sadia BAGAGNAN^1^, My Dung JUSSELME^1*^, Vanessa ALPHONSE^1^, Sabrina GUERIN-RECHDAOUI^2^, Anthony MARCONI^2^, Vincent ROCHER^2^ and Régis MOILLERON^1^

^1^Leesu, Univ Paris Est Creteil, Ecole des Ponts, Creteil, France

^2^SIAAP, Direction de l’Innovation, F-92700 Colombes, France

*************************************************************************

***Corresponding author:**

Email: [jusselme@u-pec.fr](mailto:jusselme@u-pec.fr)

Full postal address:

Laboratoire Eau Environnement et Systèmes Urbains (Leesu)

Univ Paris Est Creteil

61 avenue du Général de Gaulle, 94000-Créteil, France

Table S1: Predicted functions of level 2 microbial communities as a function of CTs

| **Level 1** | **Level 2** | CT (mg/L•min) | | | | | | |
| --- | --- | --- | --- | --- | --- | --- | --- | --- |
|  |  | **0** | **8** | **20** | **40** | **48** | **120** | **240** |
| Cellular Processes | Cell growth and death | 1.32 | 1.33 | 1.32 | 1.31 | 1.32 | 1.32 | 1.30 |
|  | Cell motility | 2.90 | 2.68 | 2.57 | 2.68 | 2.54 | 2.86 | 2.69 |
|  | Cellular community - eukaryotes | 0.00 | 0.00 | 0.00 | 0.00 | 0.00 | 0.00 | 0.00 |
|  | Cellular community - prokaryotes | 0.25 | 0.22 | 0.21 | 0.21 | 0.21 | 0.23 | 0.21 |
|  | Transport and catabolism | 0.48 | 0.45 | 0.45 | 0.44 | 0.45 | 0.45 | 0.45 |
| Environmental Information Processing | Membrane transport | 1.96 | 1.97 | 1.96 | 2.04 | 1.98 | 2.06 | 2.06 |
|  | Signal transduction | 0.67 | 0.64 | 0.62 | 0.66 | 0.63 | 0.68 | 0.67 |
| Genetic Information Processing | Folding, sorting and degradation | 2.82 | 2.81 | 2.82 | 2.78 | 2.83 | 2.80 | 2.78 |
|  | Replication and repair | 4.84 | 4.82 | 4.86 | 4.66 | 4.66 | 4.72 | 4.65 |
|  | Transcription | 0.75 | 0.75 | 0.75 | 0.72 | 0.75 | 0.74 | 0.72 |
|  | Translation | 2.23 | 2.30 | 2.32 | 2.24 | 2.33 | 2.25 | 2.23 |
| Human Diseases | Cancer: overview | 0.04 | 0.04 | 0.04 | 0.04 | 0.04 | 0.04 | 0.04 |
|  | Cardiovascular disease | 0.13 | 0.13 | 0.12 | 0.13 | 0.12 | 0.13 | 0.13 |
|  | Endocrine and metabolic disease | 0.00 | 0.00 | 0.00 | 0.15 | 0.00 | 0.00 | 0.00 |
|  | Infectious disease: bacterial | 0.12 | 0.12 | 0.12 | 0.12 | 0.12 | 0.12 | 0.12 |
|  | Infectious disease: parasitic | 0.14 | 0.12 | 0.12 | 0.13 | 0.12 | 0.13 | 0.13 |
|  | Neurodegenerative disease | 0.43 | 0.45 | 0.44 | 0.46 | 0.43 | 0.46 | 0.42 |
| Metabolism | Amino acid metabolism | 13.54 | 13.21 | 13.24 | 13.40 | 13.26 | 13.34 | 13.42 |
|  | Biosynthesis of other secondary metabolites | 2.90 | 2.91 | 2.90 | 2.83 | 2.91 | 2.87 | 2.83 |
|  | Carbohydrate metabolism | 13.10 | 13.24 | 13.23 | 13.13 | 13.19 | 13.12 | 13.16 |
|  | Energy metabolism | 5.29 | 5.32 | 5.34 | 5.25 | 5.34 | 5.27 | 5.24 |
|  | Glycan biosynthesis and metabolism | 4.58 | 3.32 | 3.32 | 3.08 | 3.23 | 3.11 | 3.06 |
|  | Lipid metabolism | 6.26 | 6.11 | 6.14 | 6.19 | 6.13 | 6.14 | 6.18 |
|  | Metabolism of cofactors and vitamins | 12.79 | 11.49 | 11.54 | 11.33 | 11.54 | 11.44 | 11.29 |
|  | Metabolism of other amino acids | 5.17 | 4.91 | 4.89 | 5.07 | 4.90 | 5.04 | 5.11 |
|  | Metabolism of terpenoids and polyketides | 9.14 | 9.36 | 9.47 | 9.23 | 9.52 | 9.13 | 9.27 |
|  | Nucleotide metabolism | 1.56 | 1.58 | 1.59 | 1.52 | 1.59 | 1.54 | 1.51 |
|  | Xenobiotics biodegradation and metabolism | 7.87 | 8.95 | 8.88 | 9.46 | 8.91 | 9.29 | 9.56 |
| Organismal Systems | Digestive system | 0.04 | 0.04 | 0.04 | 0.03 | 0.04 | 0.04 | 0.03 |
|  | Endocrine system | 0.38 | 0.41 | 0.40 | 0.43 | 0.41 | 0.41 | 0.43 |
|  | Environmental adaptation | 0.18 | 0.19 | 0.18 | 0.18 | 0.18 | 0.19 | 0.18 |
|  | Immune system | 0.11 | 0.10 | 0.11 | 0.10 | 0.11 | 0.10 | 0.10 |

Table S2: Predicted functions in relation to human diseases of level 3 microbial communities

| Level 1 | Level 2 | Level 3 | CT (mg/L•min) | | | | | | |
| --- | --- | --- | --- | --- | --- | --- | --- | --- | --- |
|  |  |  | **0** | **8** | **20** | **40** | **48** | **120** | **240** |
| Human Diseases | Cancer: overview | Pathways in cancer | 0.04 | 0.04 | 0.04 | 0.04 | 0.04 | 0.04 | 0.04 |
|  | Cardiovascular disease | Hypertrophic cardiomyopathy | 0.01 | 0.00 | 0.00 | 0.01 | 0.00 | 0.01 | 0.01 |
|  |  | Viral myocarditis | 0.12 | 0.13 | 0.12 | 0.13 | 0.12 | 0.12 | 0.13 |
|  | Endocrine and metabolic disease | Type I diabetes mellitus | 0.00 | 0.00 | 0.00 | 0.15 | 0.00 | 0.00 | 0.00 |
|  | Immune disease | Systemic lupus erythematosus | 0.00 | 0.01 | 0.00 | 0.00 | 0.00 | 0.01 | 0.00 |
|  | Infectious disease: parasitic | African trypanosomiasis | 0.08 | 0.05 | 0.05 | 0.06 | 0.05 | 0.06 | 0.06 |
|  |  | Amoebiasis | 0.02 | 0.02 | 0.02 | 0.02 | 0.02 | 0.02 | 0.02 |
|  |  | Toxoplasmosis | 0.05 | 0.05 | 0.05 | 0.05 | 0.05 | 0.05 | 0.05 |
|  | Neurodegenerative  disease | Alzheimer disease | 0.10 | 0.10 | 0.10 | 0.10 | 0.10 | 0.10 | 0.10 |
|  |  | Amyotrophic lateral sclerosis | 0.25 | 0.22 | 0.21 | 0.22 | 0.21 | 0.23 | 0.23 |
|  |  | Parkinson disease | 0.08 | 0.08 | 0.08 | 0.09 | 0.08 | 0.08 | 0.09 |
|  |  | Prion disease | 0.00 | 0.04 | 0.04 | 0.05 | 0.04 | 0.04 | 0.00 |
|  |  | Epithelial cell signalling in *Helicobacter pylori* infection | 0.09 | 0.09 | 0.09 | 0.09 | 0.09 | 0.09 | 0.09 |
|  |  | *Staphylococcus aureus* infection | 0.02 | 0.02 | 0.02 | 0.02 | 0.02 | 0.02 | 0.02 |
